# Doctoring through the Newspapers

**Published:** 1881-07

**Authors:** 


Doctoring Through the Newspapers again.—The *Live Stock Journal* has chosen to criticise our remarks in a previous issue on " Doctoring through the newspapers." If the journal in question had read our editorial more carefully it would have found that there was less difference of opinion between us than it supposes.

We did not belittle the efforts of the veterinary editors of the various stock and agricultural journals. On the contrary, we stated distinctly that so far as we had seen these journals they were ably edited, and that the best was made of bad method.

Neither did we condemn the practice of doctoring through the newspapers utterly. Such methods we said, in very much the language of our critic, must be adopted for the present, because good veterinarians are few. But we do insist that the practice is intrinsically a bad one, and ought to be abandoned as soon as it can be done with advantage. What possible accuracy in diagnosis can be obtained by the descriptions of untrained persons utterly ignorant of disease? And what certainty of treatment or what progress in veterinary science can be thus secured? We accept the practice as a necessity just now; but we assert again that veterinarians should allow it only a provisional endorsement. It will be equally for the interest of the profession and the pubilc that a veterinarian rather than the papers should be consulted whenever possible. We cannot agree either that such a book as that of Dr. Law's or of Dr. Navin's is not worth the paper it is printed on. A careful study of such works as these would often be of more value than the vague advice in veterinary departments.

